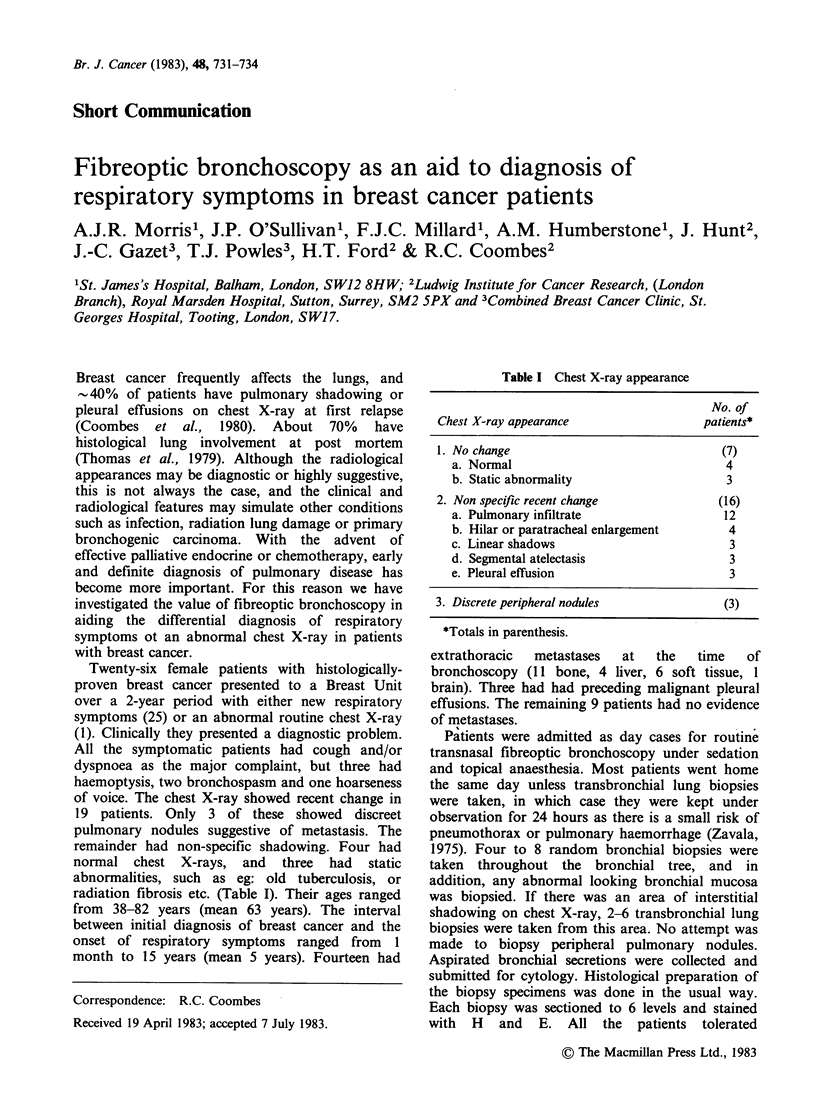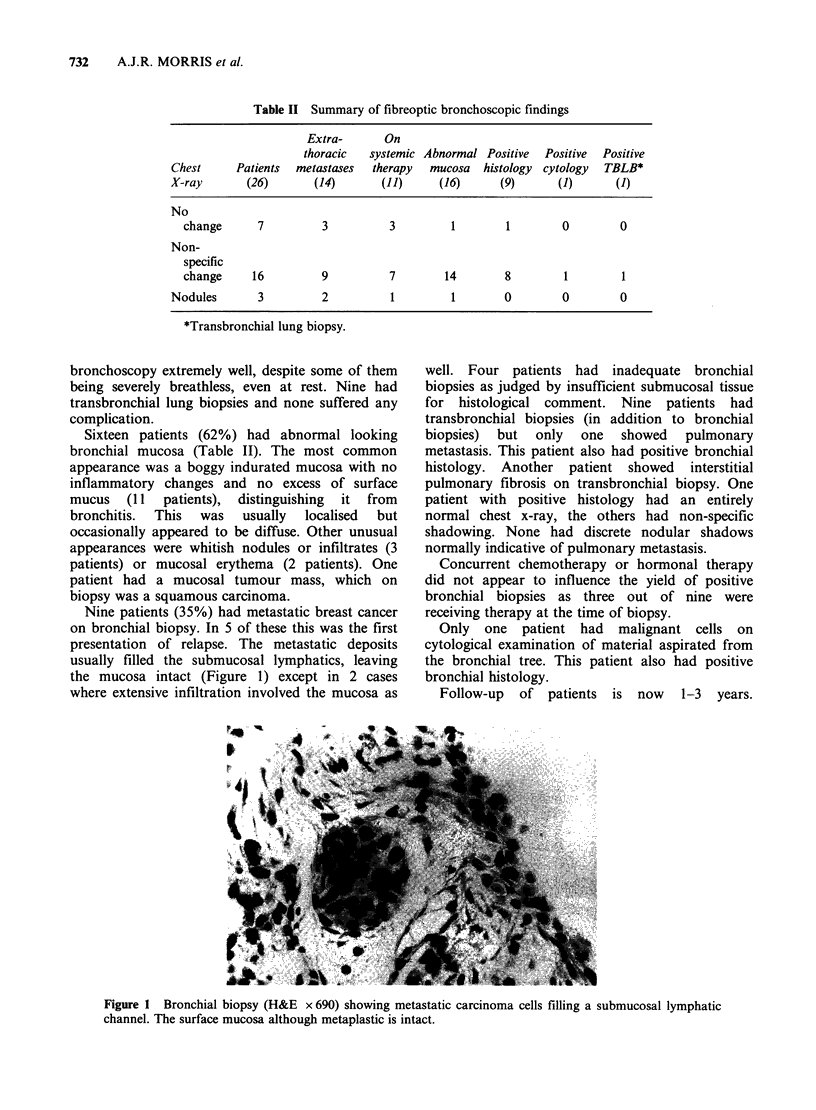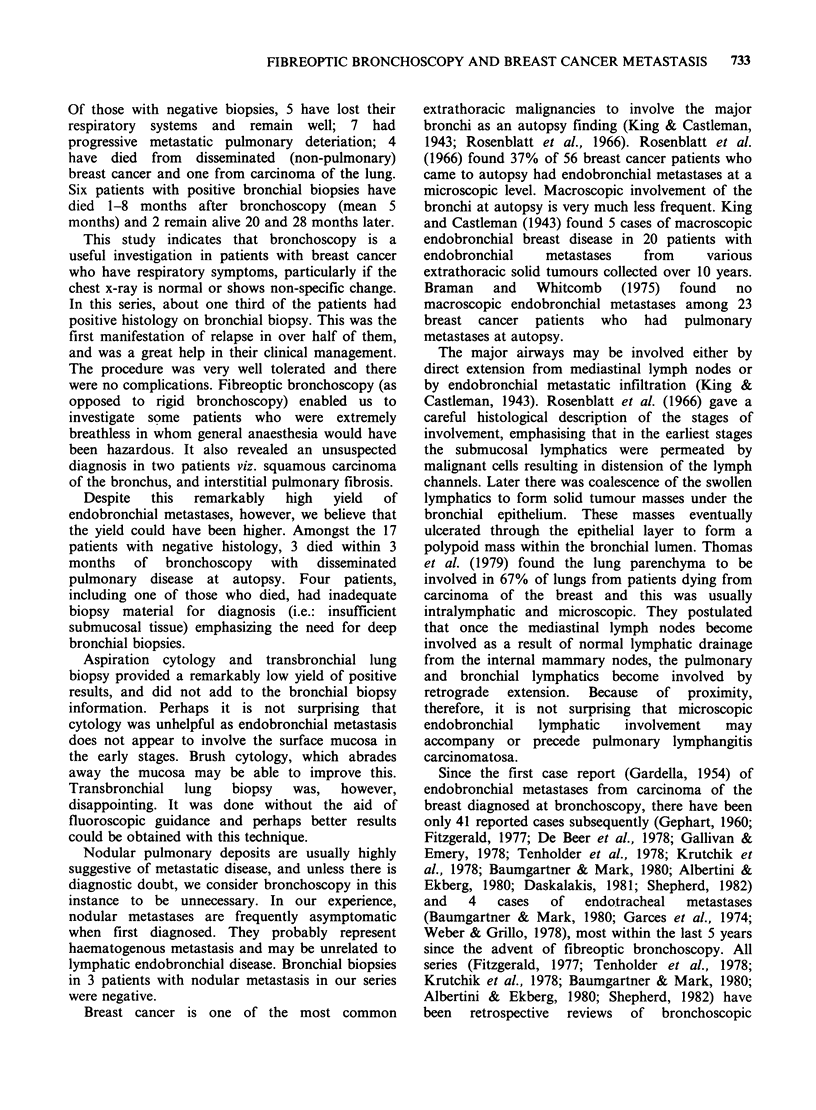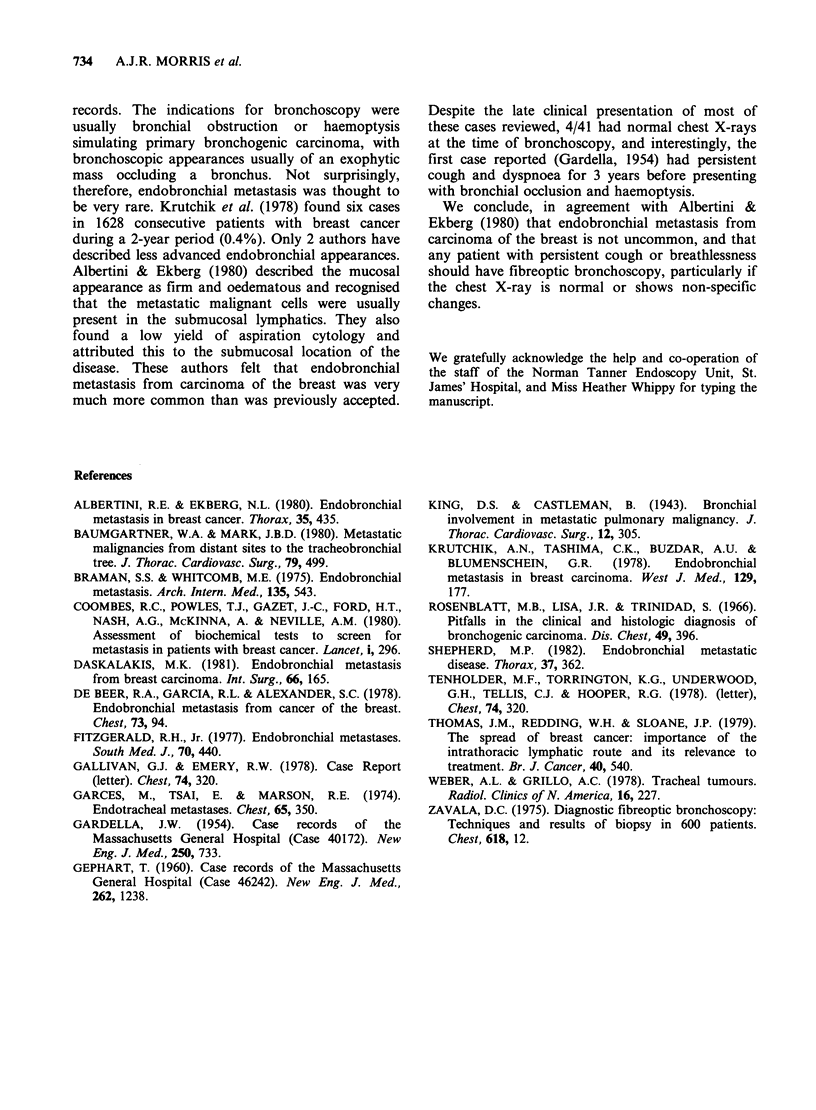# Fibreoptic bronchoscopy as an aid to diagnosis of respiratory symptoms in breast cancer patients.

**DOI:** 10.1038/bjc.1983.257

**Published:** 1983-11

**Authors:** A. J. Morris, J. P. O'Sullivan, F. J. Millard, A. M. Humberstone, J. Hunt, J. C. Gazet, T. J. Powles, H. T. Ford, R. C. Coombes

## Abstract

**Images:**


*Br. J. Cancer* (1983), **48**, 731–734

### Short Communication

### Fibreoptic bronchoscopy as an aid to diagnosis of respiratory symptoms in breast cancer patients

A.J.R. Morris[1], J.P. O'Sullivan[1], F.J.C. Millard[1], A.M. Humberstone[1], J. Hunt[2], J.-C. Gazet[3], T.J. Powles[3], H.T. Ford[2] & R.C. Coombes[2]

[1]*St. James's Hospital, Balham, London, SW12 8HW;* [2]*Ludwig Institute for Cancer Research, (London Branch), Royal Marsden Hospital, Sutton, Surrey, SM2 5PX and* [3]*Combined Breast Cancer Clinic, St. Georges Hospital, Tooting, London, SW17.*

Breast cancer frequently affects the lungs, and ~40% of patients have pulmonary shadowing or pleural effusions on chest X-ray at first relapse (Coombes *et al.,* 1980). About 70% have histological lung involvement at post mortem (Thomas *et al.,* 1979). Although the radiological appearances may be diagnostic or highly suggestive, this is not always the case, and the clinical and radiological features may simulate other conditions such as infection, radiation lung damage or primary bronchogenic carcinoma. With the advent of effective palliative endocrine or chemotherapy, early and definite diagnosis of pulmonary disease has become more important. For this reason we have investigated the value of fibreoptic bronchoscopy in aiding the differential diagnosis of respiratory symptoms ot an abnormal chest X-ray in patients with breast cancer.

Twenty-six female patients with histologically-proven breast cancer presented to a Breast Unit over a 2-year period with either new respiratory symptoms (25) or an abnormal routine chest X-ray (1). Clinically they presented a diagnostic problem. All the symptomatic patients had cough and/or dyspnoea as the major complaint, but three had haemoptysis, two bronchospasm and one hoarseness of voice. The chest X-ray showed recent change in 19 patients. Only 3 of these showed discreet pulmonary nodules suggestive of metastasis. The remainder had non-specific shadowing. Four had normal chest X-rays, and three had static abnormalities, such as eg: old tuberculosis, or radiation fibrosis etc. (Table I). Their ages ranged from 38–82 years (mean 63 years). The interval between initial diagnosis of breast cancer and the onset of respiratory symptoms ranged from 1 month to 15 years (mean 5 years). Fourteen had extrathoracic metastases at the time of bronchoscopy (11 bone, 4 liver, 6 soft tissue, 1 brain). Three had had preceding malignant pleural effusions. The remaining 9 patients had no evidence of metastases.

**Table I** Chest X-ray appearance

| *Chest X-ray appearance* | *No. of patients** |
|---|---|
| 1. *No change* | (7) |
| a. Normal | 4 |
| b. Static abnormality | 3 |
| 2. *Non specific recent change* | (16) |
| a. Pulmonary infiltrate | 12 |
| b. Hilar or paratracheal enlargement | 4 |
| c. Linear shadows | 3 |
| d. Segmental atelectasis | 3 |
| e. Pleural effusion | 3 |
| 3. *Discrete peripheral nodules* | (3) |

*Totals in parenthesis.

Patients were admitted as day cases for routine transnasal fibreoptic bronchoscopy under sedation and topical anaesthesia. Most patients went home the same day unless transbronchial lung biopsies were taken, in which case they were kept under observation for 24 hours as there is a small risk of pneumothorax or pulmonary haemorrhage (Zavala, 1975). Four to 8 random bronchial biopsies were taken throughout the bronchial tree, and in addition, any abnormal looking bronchial mucosa was biopsied. If there was an area of interstitial shadowing on chest X-ray, 2–6 transbronchial lung biopsies were taken from this area. No attempt was made to biopsy peripheral pulmonary nodules. Aspirated bronchial secretions were collected and submitted for cytology. Histological preparation of the biopsy specimens was done in the usual way. Each biopsy was sectioned to 6 levels and stained with H and E. All the patients tolerated

Correspondence: R.C. Coombes

Received 19 April 1983; accepted 7 July 1983.

© The Macmillan Press Ltd., 1983

**Table II** Summary of fibreoptic bronchoscopic findings

| *Chest X-ray* | *Patients (26)* | *Extra-thoracic metastases (14)* | *On systemic therapy (11)* | *Abnormal mucosa (16)* | *Positive histology (9)* | *Positive cytology (1)* | *Positive TBLB* (1)* |
|---|---|---|---|---|---|---|---|
| No change | 7 | 3 | 3 | 1 | 1 | 0 | 0 |
| Non-specific change | 16 | 9 | 7 | 14 | 8 | 1 | 1 |
| Nodules | 3 | 2 | 1 | 1 | 0 | 0 | 0 |

*Transbronchial lung biopsy.

bronchoscopy extremely well, despite some of them being severely breathless, even at rest. Nine had transbronchial lung biopsies and none suffered any complication.

Sixteen patients (62%) had abnormal looking bronchial mucosa (Table II). The most common appearance was a boggy indurated mucosa with no inflammatory changes and no excess of surface mucus (11 patients), distinguishing it from bronchitis. This was usually localised but occasionally appeared to be diffuse. Other unusual appearances were whitish nodules or infiltrates (3 patients) or mucosal erythema (2 patients). One patient had a mucosal tumour mass, which on biopsy was a squamous carcinoma.

Nine patients (35%) had metastatic breast cancer on bronchial biopsy. In 5 of these this was the first presentation of relapse. The metastatic deposits usually filled the submucosal lymphatics, leaving the mucosa intact (Figure 1) except in 2 cases where extensive infiltration involved the mucosa as well. Four patients had inadequate bronchial biopsies as judged by insufficient submucosal tissue for histological comment. Nine patients had transbronchial biopsies (in addition to bronchial biopsies) but only one showed pulmonary metastasis. This patient also had positive bronchial histology. Another patient showed interstitial pulmonary fibrosis on transbronchial biopsy. One patient with positive histology had an entirely normal chest x-ray, the others had non-specific shadowing. None had discrete nodular shadows normally indicative of pulmonary metastasis.

Concurrent chemotherapy or hormonal therapy did not appear to influence the yield of positive bronchial biopsies as three out of nine were receiving therapy at the time of biopsy.

Only one patient had malignant cells on cytological examination of material aspirated from the bronchial tree. This patient also had positive bronchial histology.

Follow-up of patients is now 1–3 years.

**Figure 1** Bronchial biopsy (H&E ×690) showing metastatic carcinoma cells filling a submucosal lymphatic channel. The surface mucosa although metaplastic is intact.

Of those with negative biopsies, 5 have lost their respiratory systems and remain well; 7 had progressive metastatic pulmonary deteriation; 4 have died from disseminated (non-pulmonary) breast cancer and one from carcinoma of the lung. Six patients with positive bronchial biopsies have died 1–8 months after bronchoscopy (mean 5 months) and 2 remain alive 20 and 28 months later.

This study indicates that bronchoscopy is a useful investigation in patients with breast cancer who have respiratory symptoms, particularly if the chest x-ray is normal or shows non-specific change. In this series, about one third of the patients had positive histology on bronchial biopsy. This was the first manifestation of relapse in over half of them, and was a great help in their clinical management. The procedure was very well tolerated and there were no complications. Fibreoptic bronchoscopy (as opposed to rigid bronchoscopy) enabled us to investigate some patients who were extremely breathless in whom general anaesthesia would have been hazardous. It also revealed an unsuspected diagnosis in two patients *viz*. squamous carcinoma of the bronchus, and interstitial pulmonary fibrosis.

Despite this remarkably high yield of endobronchial metastases, however, we believe that the yield could have been higher. Amongst the 17 patients with negative histology, 3 died within 3 months of bronchoscopy with disseminated pulmonary disease at autopsy. Four patients, including one of those who died, had inadequate biopsy material for diagnosis (i.e.: insufficient submucosal tissue) emphasizing the need for deep bronchial biopsies.

Aspiration cytology and transbronchial lung biopsy provided a remarkably low yield of positive results, and did not add to the bronchial biopsy information. Perhaps it is not surprising that cytology was unhelpful as endobronchial metastasis does not appear to involve the surface mucosa in the early stages. Brush cytology, which abrades away the mucosa may be able to improve this. Transbronchial lung biopsy was, however, disappointing. It was done without the aid of fluoroscopic guidance and perhaps better results could be obtained with this technique.

Nodular pulmonary deposits are usually highly suggestive of metastatic disease, and unless there is diagnostic doubt, we consider bronchoscopy in this instance to be unnecessary. In our experience, nodular metastases are frequently asymptomatic when first diagnosed. They probably represent haematogenous metastasis and may be unrelated to lymphatic endobronchial disease. Bronchial biopsies in 3 patients with nodular metastasis in our series were negative.

Breast cancer is one of the most common extrathoracic malignancies to involve the major bronchi as an autopsy finding (King & Castleman, 1943; Rosenblatt *et al.,* 1966). Rosenblatt *et al.* (1966) found 37% of 56 breast cancer patients who came to autopsy had endobronchial metastases at a microscopic level. Macroscopic involvement of the bronchi at autopsy is very much less frequent. King and Castleman (1943) found 5 cases of macroscopic endobronchial breast disease in 20 patients with endobronchial metastases from various extrathoracic solid tumours collected over 10 years. Braman and Whitcomb (1975) found no macroscopic endobronchial metastases among 23 breast cancer patients who had pulmonary metastases at autopsy.

The major airways may be involved either by direct extension from mediastinal lymph nodes or by endobronchial metastatic infiltration (King & Castleman, 1943). Rosenblatt *et al.* (1966) gave a careful histological description of the stages of involvement, emphasising that in the earliest stages the submucosal lymphatics were permeated by malignant cells resulting in distension of the lymph channels. Later there was coalescence of the swollen lymphatics to form solid tumour masses under the bronchial epithelium. These masses eventually ulcerated through the epithelial layer to form a polypoid mass within the bronchial lumen. Thomas *et al.* (1979) found the lung parenchyma to be involved in 67% of lungs from patients dying from carcinoma of the breast and this was usually intralymphatic and microscopic. They postulated that once the mediastinal lymph nodes become involved as a result of normal lymphatic drainage from the internal mammary nodes, the pulmonary and bronchial lymphatics become involved by retrograde extension. Because of proximity, therefore, it is not surprising that microscopic endobronchial lymphatic involvement may accompany or precede pulmonary lymphangitis carcinomatosa.

Since the first case report (Gardella, 1954) of endobronchial metastases from carcinoma of the breast diagnosed at bronchoscopy, there have been only 41 reported cases subsequently (Gephart, 1960; Fitzgerald, 1977; De Beer *et al.,* 1978; Gallivan & Emery, 1978; Tenholder *et al.,* 1978; Krutchik *et al.,* 1978; Baumgartner & Mark, 1980; Albertini & Ekberg, 1980; Daskalakis, 1981; Shepherd, 1982) and 4 cases of endotracheal metastases (Baumgartner & Mark, 1980; Garces *et al.,* 1974; Weber & Grillo, 1978), most within the last 5 years since the advent of fibreoptic bronchoscopy. All series (Fitzgerald, 1977; Tenholder *et al.,* 1978; Krutchik *et al.,* 1978; Baumgartner & Mark, 1980; Albertini & Ekberg, 1980; Shepherd, 1982) have been retrospective reviews of bronchoscopic records. The indications for bronchoscopy were usually bronchial obstruction or haemoptysis simulating primary bronchogenic carcinoma, with bronchoscopic appearances usually of an exophytic mass occluding a bronchus. Not surprisingly, therefore, endobronchial metastasis was thought to be very rare. Krutchik *et al.* (1978) found six cases in 1628 consecutive patients with breast cancer during a 2-year period (0.4%). Only 2 authors have described less advanced endobronchial appearances. Albertini & Ekberg (1980) described the mucosal appearance as firm and oedematous and recognised that the metastatic malignant cells were usually present in the submucosal lymphatics. They also found a low yield of aspiration cytology and attributed this to the submucosal location of the disease. These authors felt that endobronchial metastasis from carcinoma of the breast was very much more common than was previously accepted. Despite the late clinical presentation of most of these cases reviewed, 4/41 had normal chest X-rays at the time of bronchoscopy, and interestingly, the first case reported (Gardella, 1954) had persistent cough and dyspnoea for 3 years before presenting with bronchial occlusion and haemoptysis.

We conclude, in agreement with Albertini & Ekberg (1980) that endobronchial metastasis from carcinoma of the breast is not uncommon, and that any patient with persistent cough or breathlessness should have fibreoptic bronchoscopy, particularly if the chest X-ray is normal or shows non-specific changes.

We gratefully acknowledge the help and co-operation of the staff of the Norman Tanner Endoscopy Unit, St. James' Hospital, and Miss Heather Whippy for typing the manuscript.

### References

ALBERTINI, R.E. & EKBERG, N.L. (1980). Endobronchial metastasis in breast cancer. *Thorax*, **35**, 435.

BAUMGARTNER, W.A. & MARK, J.B.D. (1980). Metastatic malignancies from distant sites to the tracheobronchial tree. *J. Thorac. Cardiovasc. Surg.*, **79**, 499.

BRAMAN, S.S. & WHITCOMB, M.E. (1975). Endobronchial metastasis. *Arch. Intern. Med.*, **135**, 543.

COOMBES, R.C., POWLES, T.J., GAZET, J.-C., FORD, H.T., NASH, A.G., McKINNA, A. & NEVILLE, A.M. (1980). Assessment of biochemical tests to screen for metastasis in patients with breast cancer. *Lancet*, **i**, 296.

DASKALAKIS, M.K. (1981). Endobronchial metastasis from breast carcinoma. *Int. Surg.*, **66**, 165.

DE BEER, R.A., GARCIA, R.L. & ALEXANDER, S.C. (1978). Endobronchial metastasis from cancer of the breast. *Chest*, **73**, 94.

FITZGERALD, R.H., Jr. (1977). Endobronchial metastases. *South Med. J.*, **70**, 440.

GALLIVAN, G.J. & EMERY, R.W. (1978). Case Report (letter). *Chest*, **74**, 320.

GARCES, M., TSAI, E. & MARSON, R.E. (1974). Endotracheal metastases. *Chest*, **65**, 350.

GARDELLA, J.W. (1954). Case records of the Massachusetts General Hospital (Case 40172). *New Eng. J. Med.*, **250**, 733.

GEPHART, T. (1960). Case records of the Massachusetts General Hospital (Case 46242). *New Eng. J. Med.*, **262**, 1238.

KING, D.S. & CASTLEMAN, B. (1943). Bronchial involvement in metastatic pulmonary malignancy. *J. Thorac. Cardiovasc. Surg.*, **12**, 305.

KRUTCHIK, A.N., TASHIMA, C.K., BUZDAR, A.U. & BLUMENSCHEIN, G.R. (1978). Endobronchial metastasis in breast carcinoma. *West J. Med.*, **129**, 177.

ROSENBLATT, M.B., LISA, J.R. & TRINIDAD, S. (1966). Pitfalls in the clinical and histologic diagnosis of bronchogenic carcinoma. *Dis. Chest*, **49**, 396.

SHEPHERD, M.P. (1982). Endobronchial metastatic disease. *Thorax*, **37**, 362.

TENHOLDER, M.F., TORRINGTON, K.G., UNDERWOOD, G.H., TELLIS, C.J. & HOOPER, R.G. (1978). (letter), *Chest*, **74**, 320.

THOMAS, J.M., REDDING, W.H. & SLOANE, J.P. (1979). The spread of breast cancer: importance of the intrathoracic lymphatic route and its relevance to treatment. *Br. J. Cancer*, **40**, 540.

WEBER, A.L. & GRILLO, A.C. (1978). Tracheal tumours. *Radiol. Clinics of N. America*, **16**, 227.

ZAVALA, D.C. (1975). Diagnostic fibreoptic bronchoscopy: Techniques and results of biopsy in 600 patients. *Chest*, **618**, 12.